# Relationship Between Scheimpflug-Based Ocular Biomechanics and Myopia Progression in Adolescents

**DOI:** 10.3390/bioengineering13060615

**Published:** 2026-05-25

**Authors:** Pedro M. L. Baptista, João H. Marques, André Ferreira, Gabriel Santos, Paulo Sousa, Ricardo Parreira, Renato Ambrósio, Pedro M. A. M. Menéres, João N. M. Beirão

**Affiliations:** 1 Ophthalmology Department, Centro Hospitalar Universitário do Porto, 4099-001 Porto, Portugal; 2 Instituto de Ciências Biomédicas Abel Salazar, Universidade do Porto, 4000-008 Porto, Portugal; 3 Faculdade de Medicina da Universidade do Porto, Universidade do Porto, 4000-008 Porto, Portugal; 4 Rio de Janeiro Corneal Tomography and Biomechanics Study Group, Rio de Janeiro 20420-040, Brazil; 5 Department of Cornea and Refractive Surgery, Instituto de Olhos Renato Ambrósio, Rio de Janeiro 20420-040, Brazil; 6 Department of Opthalmology, Federal University of the State of Rio de Janeiro (UNIRIO), Rio de Janeiro 20270-004, Brazil; 7 Ophthalmology Department, Federal University of São Paulo (UNIFESP), São Paulo 04021-001, Brazil; 8 Brazilian Study Group of Artificial Intelligence and Corneal Analysis—BrAIN, Rio de Janeiro 20520-050, Brazil

**Keywords:** axial length, corneal biomechanics, corvis, children, emmetropization, high myopia, intraocular pressure, myopia, miopization, refraction, scheimpflug camera, sum of segments, vitreous cavity

## Abstract

Background/Objectives: To describe the progression of axial and segmental ocular biometric lengths and refractive status in adolescents and study independent associations between these changes and baseline ocular biomechanics. Methods: Prospective cohort of 126 eyes from 63 individuals followed for 2.5 years. Data from general health and lifestyle were collected through a validated questionnaire. Data from ocular biometry (IOL MASTER 700^®^), objective refraction, and ocular biomechanics (Corvis ST^®^) were collected at baseline and the end of follow-up timepoints. Biomechanical parameters were correlated with the variation in axial length (d_AL), vitreous cavity length (d_VCL), and spherical equivalent (d_SE). Multivariable linear regression models (one eye randomly assigned) adjusted for age, SE, and AL were developed to identify independent associations between baseline biomechanics and d_AL, d_VCL, and d_SE. Results: The cohort of the present work had a mean age of 14.1 ± 2.6 years at baseline. Variations of 0.122 ± 0.17 mm, 0.092 ± 0.17 mm, and −0.32 ± 0.9 D were found in AL, VCL, and SE at follow-up, respectively. Within the multivariable regression models, the biomechanical parameters found to be independently associated with d_AL (model 1), d_VCL (model 2), and d_SE (model 3) were as follows: Model 1—Biomechanically corrected IOP (bIOP), Integrated Radius (IR), and A2 Deflection Area (A2DArea); Model 2—bIOP, IR, and A2DArea; and Model 3—IR and WholeEyeMovementMAxTime (MaxWEMT). Conclusions: The study of ocular biomechanical behavior may play a pivotal role in the risk assessment of ocular elongation and myopic progression. This work found independent associations between ocular biomechanical behavior at baseline and axial and segmental ocular elongation and refractive myopization, mainly including bIOP, IR, and MaxWEMT.

## 1. Introduction

Myopia is a common condition, estimated to affect around 2.5 billion people worldwide nowadays [1,2]. Moreover, its prevalence has been increasing over recent decades, with projections suggesting that by 2050, approximately 50% of the global population will be affected, including around 10% with high myopia [2]. Indeed, various population-based studies conducted across different geographic regions in recent decades have revealed differences in growth patterns. Although historically the steepest increases have been reported in Asian countries [3], increasingly consistent data from Europe (The European Eye Epidemiology Consortium) [4], the United States (United States of America National Health and Nutrition Examination Survey) [5], Latin America [6], and Africa [7] describe the same trend.

This growing evidence that myopia is emerging as a global epidemic carries significant clinical and economic implications. On one hand, there will be an increased need for diagnosis and refractive correction, whether through clinical methods such as glasses or via refractive surgery [8]. On the other hand, the increasing prevalence of myopia also implies that complications such as retinal detachment [9], glaucoma [10], and myopic maculopathy [1], mostly seen in high myopia, will become more common, resulting in visual impairment in middle- to older-aged individuals, including a proportion of the working-age population, with consequent economic implications. A meta-analysis estimated the high costs of productivity loss related to myopia in the United States in 2015 [11]. Therefore, it is assumed that costs will continue to rise in line with increasing prevalence unless preventive strategies are implemented.

For several decades, researchers have been studying the factors associated with the rise of myopia, in an attempt to flatten its prevalence curves. It is now known that only a small proportion of myopia cases carry a strong hereditary component with early onset and generally high degrees of refractive error, with literature descriptions of chromosomal abnormalities or associated genetic mutations [12]. Conversely, the majority of myopia cases, especially in developed countries, appear during childhood and adolescence, typically during the school years, and are commonly referred to as school myopia. There is growing evidence supporting the association between the recent rise in school myopia and increasingly urban lifestyles [4,13,14,15,16,17,18], including exacerbating factors such as the COVID-19 pandemic [19], while in cases of high myopia, there is a more genetically driven disproportionate elongation of the posterior segment of the eye [20]. Despite the growing body of evidence regarding etiological factors, the causal relationships and underlying pathophysiological substrate have not yet been clearly established.

The theory of emmetropization is currently the most widely accepted general explanation for myopia [20], but the biological mechanisms underpinning it are not yet fully understood [21,22]. The pathways that modulate this complex process include genetic modulation [23] of the scleral tissue [24,25,26], but there is no data in the literature regarding the possible relationship between the mechanical behavior of ocular tissues and the way emmetropization occurs, particularly in terms of segmental eye elongation and progression of refractive error.

Today, it is possible to study the in vivo biomechanical behavior of the corneo-scleral unit by observing its movement after an air-puff, using a non-contact tonometer equipped with a Scheimpflug camera—Corvis-ST^®^ [27,28,29].

The present study aimed to describe the progression of axial and segmental ocular biometric lengths and refractive status in a cohort of Portuguese adolescents and study the independent associations between these changes and baseline ocular biomechanics.

## 2. Materials and Methods

### 2.1. Design

This is a prospective cohort study. Data was collected at 2 time points, separated by 2.5 ± 0.1 years. The study adhered to the tenets of the Declaration of Helsinki.

### 2.2. Setting

Centro Hospitalar e Universitário do Porto.

### 2.3. Inclusion and Exclusion Criteria

One-hundred and twenty-six eyes from sixty-three healthy adolescents (under 18 years old) from general ophthalmology appointments were included.

Exclusion criteria were as follows: amblyopia; any ocular surgery; the presence of corneal dystrophies or other corneal and scleral diseases; pterygium or other conjunctival diseases; any intraocular disease; the inability to fixate; phthisis bulbi or other ocular decompensated status; and cognitive inability to perform exams.

### 2.4. Demographic and Lifestyle Data

Data on age, gender, general health, and lifestyle from the follow-up interval were collected at the end of follow-up through a questionnaire (Google Form—Supplementary Materials Figure S1) accepted and validated by the Centro Hospitalar e Universitário do Porto Ethical Commission (nr 158-DEFI/160-CE). These include questions regarding familial history of myopia, history of allergies, sleeping position, ocular surface symptoms, eye-scratching habits, physical activity, sun exposure, digital devices utilization, and reading habits (Data on Supplementary Materials Table S1). Filling out the form was done by the individual, with the help of parents when necessary.

### 2.5. Ocular Biometric Data

Ocular biometric data from Axial Length (AL), segmental ocular biometric lengths (Central Corneal Thickness (CCT), Anterior Chamber Depth (ACD), and Lens Thickness (LT)), and White-To-White (W-T-W) were assessed in both timepoints by means of swept source OCT with the IOL MASTER 700^®^ (ZEISS, Oberkochen, Germany). The variable Vitreous Cavity Length (VCL) was built [VCL = AL − (CCT + ACD + LT)]. Deltas of progression were built for AL (d_AL) and VCL (d_VCL).

### 2.6. Objective Refraction Data

Non-cycloplegic objective refractive status—Sphere (S), Cylinder (C), and Spherical Equivalent (SE)—was assessed in both timepoints using the KR-800 Auto Kerato-Refractometer^®^ (TOPCON, Tokyo, Japan). The delta of progression was built for the Spherical Equivalent (d_SE).

### 2.7. Ocular Biomechanical Data

Biomechanical assessment was performed using the Corvis ST^®^ (OCULUS, Wetzlar, Germany), a non-contact tonometer equipped with a Scheimpflug camera, by analyzing Dynamic Corneal Response (DCR) parameters. Only examinations with an ‘OK’ quality score were included.

Both Corvis-derived intraocular pressure (c-IOP) and parameters from the three key timepoints were recorded: time from the start of the air puff to the first applanation (A1T), the moment of highest concavity (HCT), and the second applanation (A2T). Additional first-generation parameters were collected, including those from the maximum deformation during the oscillatory phase (Max) and Whole Eye Movement (WEM). Second-generation composite parameters, including biomechanically corrected intraocular pressure values, were also analyzed. All Scheimpflug-based parameters, along with their explanations and abbreviations used throughout this study, are summarized in Supplementary Materials Table S2.

### 2.8. Statistical Analysis

Descriptive statistics of all datasets were calculated for demographic, ocular biomechanics, ocular biometric, and objective refraction data. Normality of the data was tested with the Shapiro–Wilk and Kolmogorov–Smirnov tests.

Pearson correlation analyses were performed using one eye per subject, randomly selected, to assess the relationship between baseline biomechanical parameters and d_AL, d_VCL, and d_SE. Given the large number of comparisons, *p*-values were adjusted using the Benjamini–Hochberg false discovery rate (FDR) method to control for type I error. Both unadjusted and FDR-adjusted significance were considered in the interpretation of results.

Multivariable linear regression models were then developed to identify independent associations. Candidate predictors included baseline biomechanical parameters that showed significant or near-significant associations in univariable analyses (*p* < 0.10) and/or were considered clinically relevant. A stepwise selection procedure was applied (entry *p* < 0.05, removal *p* > 0.10) to derive the final models. To reduce the risk of multicollinearity, correlations between predictors were assessed, and variance inflation factors (VIF) were calculated, with a predefined threshold of VIF > 5 indicating collinearity. In such cases, only one of the correlated variables was retained based on clinical relevance.

Given the limited sample size (*n* = 63), the number of predictors included in each final model was restricted to maintain an appropriate ratio between sample size and model complexity. One eye per subject was randomly selected to avoid intra-subject correlation. Potential confounding by baseline age, AL, and SE was assessed by examining changes in regression coefficients after adjustment.

All analyses were performed using the SPS v26.0 and STATA software v19. All values are shown as mean ± standard deviation unless otherwise specified. All *p*-values (*p*) were 2-sided, and *p*-values < 0.05 were considered significant.

## 3. Results

The cohort of the present work had a mean age of 14.1 ± 2.6 years at the beginning of the study. The follow-up was 2.5 ± 0.1 years. There were 27 males and 36 females. Family history of myopia was present in 38%, a history of allergies was present in 43%, and 60% usually experienced ventral sleep during the follow-up. In the per-eye description, 41% of eyes were submitted to an ipsilateral sleeping position, 44% had ocular surface symptoms, and 66% were submitted to rubbing habits during the follow-up (Supplementary Materials Table S1).

The mean values of initial AL and VCL were 23.70 ± 1.2 mm and 16.57 ± 1.2 mm, with variations (deltas) of 0.122 ± 0.17 mm and 0.092 ± 0.17 mm over the follow-up, respectively. The initial SE was −1.02 ± 1.9 D, with a progression of −0.32 ± 0.9 D over the follow-up. At baseline, approximately 36% of participants presented with myopia greater than 1 diopter, and the majority (around 60%) exhibited an axial length between 23 and 25 mm, with 13.5% exceeding 25 mm (Table 1). Baseline biomechanical parameters are described in Table 2.

Within the correlations analysis between baseline biomechanics and ocular biometric and refractive variations during follow-up, several correlations with d_SE reached nominal statistical significance (*p* < 0.05). However, after FDR correction, only the association with Whole Eye Movement Time remained statistically significant. Associations with Maximum Inverse Radius and Integrated Radius showed borderline significance, while all other correlations were no longer significant after adjustment. For d_VCL, several correlations were nominally significant. However, none remained statistically significant after FDR correction for multiple comparisons. Similarly, for d_AL, several correlations reached nominal statistical significance, but none remained significant after FDR correction. All correlations are described in Table 3.

Linear regression models were used to evaluate the baseline biomechanical parameters independently associated with changes in axial length, vitreous cavity length, and spherical equivalent (Table 4). For d_AL, significant independent associations were observed for bIOP (β = 0.15, *p* = 0.021), Integrated Radius [mm^−1^] (β = −0.06, *p* = 0.005), and A2 Deflection Area [mm^2^] (β = −1.10, *p* = 0.029) (R^2^ = 0.159; adjusted R^2^ = 0.130). For d_VCL, significant independent associations were observed for bIOP (β = 0.14, *p* = 0.022), Integrated Radius [mm^−1^] (β = −0.06, *p* = 0.008), and A2 Deflection Area [mm^2^] (β = −1.12, *p* = 0.025) (R^2^ = 0.152; adjusted R^2^ = 0.122). For d_SE, significant independent associations were observed for Integrated Radius [mm^−1^] (β = 0.24, *p* = 0.018), HC Deflection Area [mm^2^] (β = −0.80, *p* = 0.003), and Whole Eye Movement Max [ms] (β = 0.42, *p* = 0.001) (R^2^ = 0.389; adjusted R^2^ = 0.357). With the inclusion of baseline age, AL and SE did not change the regression coefficients by more than 10%, so they were not considered relevant confounders after adjustment. It should be noted that the SE variable was not converted to its absolute value, so the clinical interpretation of the model’s findings is the reverse of the direction of the presented values.

## 4. Discussion

The present work describes the progression of axial and segmental ocular biometric lengths, namely axial length and vitreous cavity length, and refractive status, in a cohort of Portuguese adolescents and the independent associations between these changes and baseline ocular biomechanics.

With a mean age of 14.1 ± 2.6 years and a follow-up of 2.5 ± 0.1 years, this sample may be representative of an important phase of ocular growth and refractive changes in the life of an individual. The baseline average AL and SE of 23.7 ± 1.2 mm and −1.02 ± 1.9 D, respectively, were outside the definition of high myopia [30]. Nevertheless, AL is slightly above normal range in this cohort, and the authors of the present work place greater emphasis on the study of anatomical growth for three reasons: (1) most studies on myopic progression in school-aged children focus on refractive changes rather than axial growth; (2) there are no studies specifically focused on the evolution of vitreous cavity length and its role in myopic progression; and (3) compared to anatomical measures, refractive data have a weaker direct association with the tissue behavior that we aim to describe.

The lifestyle factors believed to be most associated with myopia progression are a high level of education [4] and a low amount of time spent outdoors [13,14,15,16], with less established evidence regarding physical activity [14,15] and the use of digital devices [17,18], including its rise with the COVID-19 pandemic [19]. As these factors are increasingly recognized as modulators of the myopization process of the human eye in today’s society, the characterization of the cohort regarding the evaluated lifestyle parameters is described in Supplementary Materials Table S1. As an objective and reproducible evaluation of such factors is the subject of another analysis from our group and is outside the scope of this work, this discussion is focused only on the independent associations between ocular biomechanics and ocular elongation and myopization.

The present work aims to understand the relationship between a given biomechanical behavior of ocular tissues during adolescence and future anatomical and refractive changes. Several studies have highlighted the potential role of tissue modulators in the sclera, particularly when examining the effect of atropine on reducing myopic progression. Although its utilization began due to its effects on accommodation [21], the paradigm has shifted. Today, it is believed that muscarinic receptors trigger tissue remodeling signaling pathways, which may be the primary effect of this drug that has been used in this setting for decades [22]. In fact, in line with the postulated idea that excessive eye growth can be due to increased scleral matrix remodeling [24], an experimental model of myopia in chicks showed that atropine treatment can make the fibrous layer of the sclera thicker and the cartilaginous layer thinner, stopping ocular growth with refractive error recovery [25].

More recently, evidence has emerged in human eyes: (1) the external application of atropine can lead to tissue modulation, even in the short term, resulting in increased scleral thickness and pressure, along with a decrease in intraocular pressure as measured at the cornea [26]; (2) there may be changes in the expression of genes involved in the repression of scleral growth after its application [23]. Based on the evidence that ocular elongation is modulated by the characteristics of the scleral tissue, the authors of this study conceptualize that the mechanical behavior of the eye can be considered a fingerprint of these characteristics. In this sense, ocular biomechanics, studied through a Scheimpflug Camera with direct visualization of tissue movement, emerges as a quick and non-invasive way to describe them [29]. However, the description of biomechanical behavior from viscoelastic tissues, such as the cornea and sclera, is complex and cannot be reduced to the analysis of most parameters in isolation [31,32]. In fact, in the correlation analysis described in Table 3, although several correlations were found between individual biomechanical parameters and the three variables under study, most remain weak after the application of the FDR method. In this regard, when the goal is to predict ocular elongation or myopic progression based on the biomechanical behavior of ocular tissues, it is crucial to seek models of in vivo ocular biomechanics that include multiple parameters, which, when analyzed individually, might theoretically be associated with biomechanical behaviors in opposite directions.

In this study, three multivariable regression models were developed to identify independent associations between ocular biomechanics and the following variables: (1) total axial elongation; (2) elongation of the vitreous cavity; and (3) refractive myopization. According to some literature, ocular biomechanics can be associated with age and AL [33,34], and these variables were therefore considered potential confounders. However, as their inclusion in the models did not modify the regression coefficients by more than 10%, they were not considered relevant confounders.

In contrast, analysis of both models related to anatomical ocular elongation demonstrated that greater total and segmental ocular elongation were independently associated with higher baseline bIOP. In line with previous studies [35,36,37,38], the authors conceptualize that younger ocular structures may be susceptible to the effects of intraocular pressure exerted internally on a more compliant sclera, in contrast to a proportionally less compliant cornea, as if it were a form of scleral ectasia. The model for refractive myopization did not include bIOP, which is understandable given that the final refraction depends on other determinants [39], such as corneal curvature and potentially ocular surface factors, which were not assessed in this study. In fact, our group is working on models including baseline tomography-derived parameters, like keratometry, corneal volume, or corneal density; however, this will be included in subsequent work, with this work remaining focused on ocular biomechanics.

Intraocular pressure is a crucial factor and deserves its own discussion. Several theories about the onset and progression of axial myopia have been described over the years [37,38], and it is believed that IOP may play a pivotal role in several of them, particularly due to its increase associated with the change in lens shape caused by the action of the ciliary muscles during sustained accommodation or due to the action exerted by the extraocular muscles during convergence involved in the near reflex. The most widely accepted rationale assumes that during this sustained reflex, IOP increases, stretching scleral fibers and leading to the cumulative remodeling over time of more susceptible sclera via feedback mechanisms, resulting in an increase in ocular length in an attempt to achieve emmetropization of the eye in response to the constant need for near vision [35,36,37]. It should be noted that the parameter included in the models is the biomechanically corrected IOP, a parameter derived from the IOP measured with the Corvis ST air-puff, to which an algorithm is applied. This algorithm has been validated as providing an IOP value independent of corneal characteristics [40] and has already been shown to be the closest to the true IOP in ex vivo experiments [41]. Its inclusion highlights the value of biomechanical study in assessing the potential effect of true IOP in the process of human eye growth.

When focusing on a purely biomechanical analysis of the models, the main findings are as follows: (1) first-generation parameters describing smaller corneal deflections (A2 Deflection Area [mm^2^]; HC Deflection Area [mm^2^]) were independently associated with greater d_AL, d_VCL, and d_SE; (2) lower values of IR were independently associated with greater d_AL and d_VCL and higher absolute d_SE; and (3) lower values of Whole Eye Movement Max [ms] were independently associated with higher absolute d_SE.

The first-generation parameters derived from the study of ocular biomechanics using a Scheimpflug camera coupled with a non-contact air-puff tonometer consist of image-based measurements of distances and areas resulting from different corneal positions observed throughout its full excursion. The models described above included measurements of corneal deflection at the point of highest concavity, at the moment of maximum deformation amplitude, and during the second corneal applanation. Overall, the findings suggested that a smaller corneal deflection amplitude at baseline was associated with greater total axial elongation of the eye and the vitreous chamber during follow-up. As corneal deflection movement represents the primary moment for dissipating the energy of cumulative microtrauma throughout life, the authors postulate, as a possible mechanism, that these corneas may have a reduced capacity for energy dissipation and, consequently, may be associated with a greater amount of cumulative energy transmitted to the posterior pole, resulting in subsequent ocular elongation. Although conceptual, this interpretation is consistent with previously published evidence [42].

It is important to understand what the other included parameters represent. The IR (second-generation parameter) is a mathematical function that represents the inverse value of the corneal radius of curvature throughout its entire movement, expressed as the area under the curve, thus giving a more consistent quantification of the cumulative corneal deflection throughout the entire movement [29]. Therefore, the inclusion of this parameter in all three models reinforces the rationale previously described, including both anatomical elongation and refractive myopization. Whole Eye Movement (WEM) is characterized by the length and duration and refers to the antero-posterior excursion that the cornea exhibits in the most lateral part of each side of the 8 mm Scheimpflug image. Although not proven yet, the authors believe that the WEM could be of great value in the study of posterior pole pathology in myopic eyes, as it is assumed to describe the accessorial movement occurring in the rest of the eye after the transmission of energy that the cornea could not absorb in its deflection movement [43].

In the present study, the authors particularly emphasize the significance of the findings related to MaxWEMT and IR to add to those related to bIOP. In this context, we hypothesized the following: (1) lower IR values indicate less cumulative corneal deflection after the air puff, potentially leading to greater energy transfer to the sclera; and (2) lower MaxWEMT values, which indicate a faster accessory movement of the cornea, may reflect greater scleral compliance in the absorption of that energy. Since the corneo-scleral unit is a functional continuum unit [44,45], its biomechanical behavior may amplify the previously described effect of higher IOP on scleral fibers and substantiate the theories related to scleral feedback in anatomical elongation [35,36,37]. Although these assumptions are theoretical and require external validation, the authors believe that the study of ocular biomechanics, particularly these results, may be part of the process of substantiating the practical effects of genetic, tissue, and behavioral factors on ocular elongation and subsequent myopization during school-aged children and are of utmost importance in a society that is preparing to face a myopia epidemic in the coming decades [2].

According to the theory that myopic growth, particularly in high myopia, occurs primarily in the posterior pole, the authors studied not only total elongation but also elongation of the vitreous cavity. The two variables did not show a linear correlation, emphasizing the value of the segmental approach. Furthermore, the significant independent associations identified between ocular biomechanics and vitreous chamber elongation in a population with a low mean myopic refractive error, such as that included in the present study, underscore the importance of investigating this parameter alongside axial length in future studies, particularly in high myopia.

The main strengths of this study are its prospective design, the follow-up of over 2.5 years in individuals during adolescence, a period recognized as particularly important in the progression of school myopia, and the combination of ocular biomechanics and both total and segmental ocular biometrics measurements and objective refraction, making it the first study in the literature to adopt this approach. Several limitations should be considered when interpreting these findings. (1) First, non-cycloplegic refraction was performed due to limitations imposed by the hospital’s ethics committee; however, this may add value to the results, as greater accommodative ability at baseline could have masked a degree of myopic progression during follow-up. (2) Second, the sample does not include only myopic eyes and includes adolescents of different ages; nevertheless, baseline age, SE, and AL were not considered relevant confounders. (3) Third, the relatively small sample size (*n* = 63) in relation to the number of candidate biomechanical variables increases the risk of overfitting. This is particularly relevant given the use of stepwise regression, which may lead to unstable variable selection and optimistic estimates of model performance. Although we attempted to mitigate this by restricting the number of predictors and assessing multicollinearity, the resulting models should not be considered robust predictive models. (4) Fourth, the absence of internal validation (e.g., bootstrapping or cross-validation) and external validation further limits the generalizability of the findings. (5) Lastly, the modest explanatory power of the models, especially for axial length and vitreous cavity length (adjusted R^2^ ≤ 0.15), indicates that a substantial proportion of variability remains unexplained. Accordingly, these results should be interpreted as exploratory and hypothesis-generating, reflecting associations between baseline biomechanical parameters and ocular changes rather than clinically applicable prediction models.

## 5. Conclusions

The study of ocular biomechanical behavior may play a pivotal role in the risk assessment of ocular elongation and myopic progression. This prospective study found independent associations between ocular biomechanical behavior at baseline and axial and segmental ocular elongation and refractive myopization. The main biomechanical parameters were the Biomechanically corrected Intraocular Pressure, the Integrated Radius, and Whole Eye Movement Max Time.

## Figures and Tables

**Table 1:** Biometric and objective refraction data (all sample: 63 individuals/126 eyes).

Biometric Data	Mean	SD
Baseline AL (mm)	23.702	1.233
Baseline VCL (mm)	16.572	1.193
Baseline CCT (mm)	0.534	0.036
Baseline ACD (mm)	3.130	0.211
Baseline LT (mm)	3.498	0.178
Baseline W-T-W (mm)	12.099	0.559
Final AL (mm)	23.824	1.303
Final VCL (mm)	16.649	1.260
Final CCT (mm)	0.543	0.037
Final ACD (mm)	3.090	0.230
Final LT (mm)	3.542	0.192
Final W-T-W (mm)	12.110	0.552
d_AL (mm)	0.122	0.170
d_VCL (mm)	0.092	0.167
Baseline AL (mm)—stratification	*n*	%
<23 mm	32	25.4
23 to 25 mm	77	61.1
>25 mm	17	13.5
Objective refraction data	Mean	SD
Baseline S (D)	−0.784	1.722
Baseline C (D)	−0.470	0.796
Baseline SE (D)	−1.020	1.885
Final S (D)	−1.008	2.054
Final C (D)	−0.627	0.902
Final SE (D)	−1.322	2.240
d_SE (D)	−0.321	0.869
Baseline SE (D)—stratification	*n*	%
<(-) 1 D	45	35.7
(-) 1.00 to 0 D	41	32.5
>0 D	40	31.7

Footnotes: AL: Axial Length; VCL: Vitreous Cavity Length; CCT: Central Corneal Thickness; LT: Lens Thickness; W-T-W: White-To-White; S: Sphere; C: Cylinder: SE: Spherical Equivalent; D: Diopters; d: delta of variation.

**Table 2:** Baseline Ocular biomechanics data (all sample: 63 individuals/126 eyes).

Ocular Biomechanics Parameter (Baseline)	Mean	Std. Deviation
cIOP [mmHg]	14.198	2.402
cCCT [µm]	554.629	36.431
Deformation Amp. Max [mm]	1.048	0.084
A1 Time [ms]	7.654	0.294
A1 Velocity [m/s]	0.143	0.016
A2 Time [ms]	22.334	0.369
A2 Velocity [m/s]	−0.264	0.029
HC Time [ms]	17.684	0.479
Peak Dist. [mm]	4.938	0.249
Radius [mm]	6.583	0.606
A1 Deformation Amp. [mm]	0.139	0.012
HC Deformation Amp. [mm]	1.048	0.084
A2 Deformation Amp. [mm]	0.357	0.057
A1 Deflection Length [mm]	2.228	0.217
HC Deflection Length [mm]	6.409	0.372
A2 Deflection Length [mm]	3.132	0.699
A1 Deflection Amp. [mm]	0.091	0.012
HC Deflection Amp. [mm]	1.014	0.242
A2 Deflection Amp. [mm]	0.109	0.014
Deflection Amp. Max [mm]	1.236	0.471
Deflection Amp. Max [ms]	16.885	0.725
Whole Eye Movement Max [mm]	0.258	0.072
Whole Eye Movement Max [ms]	21.982	0.798
A1 Deflection Area [mm^2^]	0.163	0.025
HC Deflection Area [mm^2^]	3.078	0.433
A2 Deflection Area [mm^2^]	0.25	0.05
A1 dArc Length [mm]	−0.017	0.005
HC dArc Length [mm]	−0.12	0.02
A2 dArc Length [mm]	−0.023	0.008
dArcLengthMax [mm]	−0.139	0.02
Max InverseRadius [mm^−1^]	0.185	0.023
DA Ratio Max (2 mm)	4.092	0.447
PachySlope [µm]	38.025	8.648
DA Ratio Max (1 mm)	1.535	0.053
Ambrosio Relational Thickness (8 mm)	656.1	183.457
Biomechanically-corrected IOP	14.149	1.955
Integrated Radius [mm^−1^]	9.003	1.129
Stiffness parameter in A1	101.031	19.755
Corvis biomechanical index	0.268	0.243
Tomographic and Biomechanical Index	0.197	0.224
Stress Strain Index	0.949	0.13
A1 DeflectionVelocity [m/s]	0.138	0.037
A2 DeflectionVelocity [m/s]	−0.344	0.067
Corrected Corvis biomechanical index	0.09	0.199
Biomechanically-corrected IOP (2nd version)	14.392	3.143
Stress Strain Index (2nd version)	0.731	0.152
Stiffness parameter in HC	12.386	3.93
Stiffness parameter in MaxDT	4.444	2.407

**Table 3:** Pearson´s correlations between baseline ocular biomechanical parameters and variation of Axial Length, Vitreous Cavity Length, and Spherical Equivalent during follow-up (63 eyes/63 individuals).

Ocular Biomechanics Parameter (Baseline)	Pearson’s Correlations Between d_AL and Baseline Biomechanics			Pearson’s Correlations Between d_VCL and Baseline Biomechanics			Pearson’s Correlations Between d_SE and Baseline Biomechanics		
		Pearson’s r	*p*		Pearson’s r	*p*		Pearson’s r	*p*
cIOP [mmHg]	d_AL	−0.156	0.190	d_VCL	0.142	0.133	d_SE	0.144	0.195
cCCT [µm]	d_AL	0.124	0.336	d_VCL	0.116	0.373	d_SE	0.010	0.938
Deformation Amp. Max [mm]	d_AL	−0.198	0.123	d_VCL	−0.227	0.078	d_SE	−0.011	0.933
A1 Time [ms]	d_AL	0.183	0.155	d_VCL	0.197	0.129	d_SE	−0.100	0.441
A1 Velocity [m/s]	d_AL	−0.370	0.003 **	d_VCL	−0.358	0.005 **	d_SE	0.004	0.978
A2 Time [ms]	d_AL	−0.315	0.014 *	d_VCL	−0.302	0.019 *	d_SE	0.171	0.191
A2 Velocity [m/s]	d_AL	0.139	0.286	d_VCL	0.190	0.146	d_SE	0.039	0.765
HC Time [ms]	d_AL	−0.291	0.022 *	d_VCL	−0.255	0.047 *	d_SE	0.084	0.519
Peak Dist. [mm]	d_AL	−0.076	0.559	d_VCL	−0.126	0.334	d_SE	−0.137	0.294
Radius [mm]	d_AL	0.095	0.461	d_VCL	0.117	0.371	d_SE	0.004	0.975
A1 Deformation Amp. [mm]	d_AL	−0.041	0.750	d_VCL	−0.031	0.813	d_SE	−0.146	0.262
HC Deformation Amp. [mm]	d_AL	−0.198	0.123	d_VCL	−0.227	0.078	d_SE	−0.011	0.933
A2 Deformation Amp. [mm]	d_AL	−0.123	0.347	d_VCL	−0.067	0.611	d_SE	0.154	0.241
A1 Deflection Length [mm]	d_AL	−0.030	0.814	d_VCL	0.020	0.880	d_SE	−0.135	0.300
HC Deflection Length [mm]	d_AL	−0.052	0.692	d_VCL	−0.097	0.459	d_SE	−0.150	0.252
A2 Deflection Length [mm]	d_AL	−0.175	0.177	d_VCL	−0.171	0.192	d_SE	0.065	0.622
A1 Deflection Amp. [mm]	d_AL	−0.102	0.430	d_VCL	−0.062	0.636	d_SE	−0.095	0.466
HC Deflection Amp. [mm]	d_AL	−0.117	0.365	d_VCL	−0.173	0.183	d_SE	−0.114	0.381
A2 Deflection Amp. [mm]	d_AL	−0.082	0.530	d_VCL	−0.095	0.468	d_SE	−0.062	0.637
Deflection Amp. Max [mm]	d_AL	−0.140	0.276	d_VCL	−0.193	0.137	d_SE	−0.101	0.437
Deflection Amp. Max [ms]	d_AL	−0.070	0.588	d_VCL	0.001	0.996	d_SE	0.113	0.386
Whole Eye Movement Max [mm]	d_AL	−0.113	0.383	d_VCL	−0.054	0.681	d_SE	0.148	0.257
Whole Eye Movement Max [ms]	d_AL	−0.289	0.023 *	d_VCL	−0.265	0.039 *	d_SE	0.514	<0.001 ***
A1 Deflection Area [mm^2^]	d_AL	−0.113	0.382	d_VCL	−0.093	0.476	d_SE	0.028	0.832
HC Deflection Area [mm^2^]	d_AL	−0.109	0.398	d_VCL	−0.165	0.203	d_SE	−0.166	0.201
A2 Deflection Area [mm^2^]	d_AL	−0.184	0.156	d_VCL	−0.201	0.123	d_SE	0.004	0.977
A1 dArc Length [mm]	d_AL	0.025	0.844	d_VCL	−0.016	0.902	d_SE	0.268	0.037 *
HC dArc Length [mm]	d_AL	−0.011	0.936	d_VCL	−0.008	0.951	d_SE	0.113	0.385
A2 dArc Length [mm]	d_AL	0.148	0.254	d_VCL	0.153	0.243	d_SE	0.107	0.416
dArcLengthMax [mm]	d_AL	0.080	0.538	d_VCL	0.084	0.521	d_SE	−0.034	0.794
Max InverseRadius [mm^−1^]	d_AL	−0.053	0.685	d_VCL	−0.011	0.934	d_SE	0.369	0.003 **
DA Ratio Max (2 mm)	d_AL	−0.241	0.060	d_VCL	−0.232	0.072	d_SE	0.063	0.628
PachySlope [µm]	d_AL	−0.096	0.456	d_VCL	−0.090	0.491	d_SE	0.143	0.272
DA Ratio Max (1 mm)	d_AL	−0.137	0.288	d_VCL	−0.127	0.329	d_SE	0.205	0.113
Ambrosio Relational Thickness (8 mm)	d_AL	−0.045	0.731	d_VCL	−0.073	0.575	d_SE	−0.272	0.034 *
Biomechanically-corrected IOP	d_AL	0.161	0.211	d_VCL	0.187	0.149	d_SE	−0.112	0.393
Integrated Radius [mm^−1^]	d_AL	−0.240	0.061	d_VCL	−0.199	0.125	d_SE	0.364	0.004 **
Stiffness parameter in A1	d_AL	0.286	0.024 *	d_VCL	0.269	0.036 *	d_SE	−0.014	0.914
Corvis biomechanical index	d_AL	−0.132	0.312	d_VCL	−0.133	0.312	d_SE	0.187	0.152
Tomographic and Biomechanical Index	d_AL	−0.109	0.403	d_VCL	−0.174	0.185	d_SE	−0.012	0.928
Stress Strain Index	d_AL	0.073	0.575	d_VCL	0.119	0.360	d_SE	0.025	0.850
A1 DeflectionVelocity [m/s]	d_AL	−0.313	0.013 *	d_VCL	−0.281	0.028 *	d_SE	0.019	0.882
A2 DeflectionVelocity [m/s]	d_AL	0.092	0.483	d_VCL	0.144	0.272	d_SE	0.001	0.994
Corrected Corvis biomechanical index	d_AL	−0.084	0.519	d_VCL	−0.097	0.463	d_SE	0.331	0.010 **
Biomechanically-corrected IOP (2nd version)	d_AL	0.043	0.738	d_VCL	0.084	0.520	d_SE	0.020	0.877
Stress Strain Index (2nd version)	d_AL	0.011	0.933	d_VCL	0.001	0.995	d_SE	−0.059	0.651
Stiffness parameter in HC	d_AL	0.155	0.230	d_VCL	0.172	0.186	d_SE	0.008	0.952
Stiffness parameter in MaxDT	d_AL	0.183	0.159	d_VCL	0.181	0.167	d_SE	−0.015	0.910

Footnotes: * *p* < 0.05, ** *p* < 0.01, *** *p* < 0.001.

**Table 4:** Linear regression models for the prediction of variation of axial length, vitreous cavity length and spherical equivalent during follow-up (63 eyes/63 individuals).

Baseline Variable	Variation of Axial Length (d_AL)					
	Coef.	Std. Err.	t	P > t	[95% Conf.	Interval]
bIOP	0.1466312	0.0383254	2.87	0.021	0.0255482	0.2275242
Integrated Radius [mm^−1^]	−0.062442	0.0213019	−2.93	0.005	−0.1050824	−0.0198015
A2 Deflection Area [mm^2^]	−1.096666	0.4909217	−2.23	0.029	−2.079353	−0.1139798
CONSTANT	0.9415013	0.2461661	3.82	0.000	0.4487464	1.434256
	R^2^ = 0.1585			Adjusted R^2^ = 0.1295		
Baseline variable	Variation of Vitreous Cavity Length (d_VCL)					
	Coef.	Std. Err.	t	P > t	[95% Conf.	Interval]
bIOP	0.1392412	0.0527198	2.79	0.022	0.0453598	0.2657611
Integrated Radius [mm^−1^]	−0.0578271	0.0211274	−2.74	0.008	−0.100134	−0.0155203
A2 Deflection Area [mm^2^]	−1.122188	0.4874765	−2.30	0.025	−2.098343	−0.1460329
CONSTANT	0.8752968	0.2443687	3.58	0.001	0.3859568	1.364637
	R^2^ = 0.1519			Adjusted R^2^ = 0.1221		
Baseline variable	Variation of Spherical Equivalent (d_SE)					
	Coef.	Std. Err.	t	P > t	[95% Conf.	Interval]
Integrated Radius [mm^−1^]	0.2403174	0.0983797	2.44	0.018	0.0433155	0.4373193
HC Deflection Area [mm^2^]	0.8034783	0.2589131	3.10	0.003	0.2850138	1.321943
WholeEyeMovementMax [ms]	0.4209958	0.1149825	3.66	0.001	0.1907472	0.6512444
CONSTANT	−9.342914	2.335435	−4.00	0.000	−14.01954	−4.666285
	R^2^ = 0.3888			Adjusted R^2^ = 0.3566		

## Data Availability

The datasets generated during and/or analyzed during the current study are available from the corresponding author on reasonable request.

## Supplementary Materials

The following supporting information can be downloaded at: https://www.mdpi.com/article/10.3390/bioengineering13060615/s1, Figure S1: Google form questionnaire—Oporto Myopia Study Questionnaire; Table S1: Demographic and lifestyle data; Table S2: Scheimpflug camera-derived corneal biomechanical parameters with explanation and abbreviations.
