# Supplementary material for: Relationship Between Scheimpflug-Based Ocular Biomechanics and Myopia Progression in Adolescents
Source: Bioengineering (Basel). 2026 May 25;13(6):615. doi: 10.3390/bioengineering13060615 (PMC13295369; doi:10.3390/bioengineering13060615)
Supplement: Supplementary file 1 [file bioengineering-13-00615-s001.zip › Figure S1 Google form questionnaire - Oporto Myopia Study Questionnaire.pdf]

QUESTIONÁRIO: *OPORTO MYOPIA STUDY*

Este é um questionário constituído por **38 questões** de resposta **simples e rápida** (**3-5 minutos**)

O objetivo é a sua utilização para **fins estatísticos**, constituindo-se desta forma como mais uma ferramenta de melhoria constante da prática clínica e da saúde ocular dos utentes do Serviço de Oftalmologia do Centro Hospitalar e Universitário de Santo António.

Todos os dados serão analisados de forma **anonimizada**, apenas para efeitos estatísticos, sem individualização das respostas.

Divide-se em **4 partes** (em cada página é apresentada 1 das partes):
- DADOS DEMOGRÁFICOS GERAIS - 8 questões
- DADOS DE SAUDE GERAL - 4 questões
- DADOS DE SAUDE OCULAR - 13 questões
- DADOS SOBRE ESTILO DE VIDA - 13 questões

Pode avançar ou retroceder entre as diferentes páginas **sem perder as respostas já dadas**

As questões estão divididas em:
- escolha múltipla **(círculos)** - escolha obrigatória de uma resposta
- escolha variada **(quadrados)** - pode escolher várias opções
- questões abertas **(linha)** - pode descrever da forma que pretender, com palavras ou números

Sempre que escolher a **opção "Outra"**, deve descrever qual

*Obrigatório

DADOS **DEMOGRÁFICOS GERAIS**

1. **INICIAIS** DO NOME *

2. NÚMERO DE **PROCESSO** CLÍNICO *

____

3. **SEXO** *

Marcar apenas uma oval.

- Feminino
- Masculino
- Sem identificação

4. **IDADE** *

____

5. **ALTURA** (metros) *

____

6. **PESO** (quilogramas) *

____

7. **CONCELHO** DE RESIDÊNCIA *

____

8. **CÓDIGO POSTAL** (1ºs 4 números) *

____

DADOS DE **SAÚDE GERAL**

9. HISTÓRIA DE **DOENÇAS ALÉRGICAS** (pode escolher várias opções) *

Marcar tudo o que for aplicável.

- [ ] Nenhuma
- [ ] Atopia
- [ ] Rinite
- [ ] Asma
- [ ] Dermatite
- [ ] Outra: ________________

10. HISTÓRIA DE **DOENÇAS AUTO-IMUNES OU DO TECIDO CONJUNTIVO** * (pode escolher várias opções)

Marcar tudo o que for aplicável.

- [ ] Nenhuma
- [ ] Artrite Reumatóide
- [ ] Lúpus
- [ ] Espondilites
- [ ] Doença da Tiroide
- [ ] Polimiosite / Dermatomiosite
- [ ] Sindrome de Marfan
- [ ] Pseudoxantoma Elástico
- [ ] Síndrome de Ehlers-Danlos
- [ ] Outra: ________________

11. HISTÓRIA DE **DOENÇAS GERAIS** (pode escolher várias opções) *

Marcar tudo o que for aplicável.

- [ ] Nenhuma
- [ ] Hipertensão Arterial Sistémica
- [ ] Diabetes
- [ ] Dislipidémia (aumento do colesterol ou outras gorduras)
- [ ] Doença coronária (ou história de enfarte cardiaco)
- [ ] Doença Pulmonar Obstrutiva Crónica (ou outra doença pulmonar crónica)
- [ ] Acidente Vascular Cerebral (AVC)
- [ ] Trombose Venosa (ou insuficiencia venosa periférica)
- [ ] Doença renal crónica
- [ ] Outra: ______________________

12. **SUPLEMENTOS** ALIMENTARES *

Marcar apenas uma oval.

- ( ) Sim
- ( ) Não

DADOS DE **SAÚDE OCULAR**

13. TEM **SINTOMAS** OCULARES HABITUALMENTE NO **OLHO DIREITO**? *
(pode escolher várias opções)

Marcar tudo o que for aplicável.

- [ ] Não tenho sintomas
- [ ] Prurido (comichão)
- [ ] Picadelas
- [ ] Sensação de areias
- [ ] Sensação de Olho seco
- [ ] Sensação de visão turva
- [ ] Lacrimejo
- [ ] Olho vermelho
- [ ] Secreção
- [ ] Fotofobia (grande desconforto com a luz)
- [ ] Visão turva em certos momentos do dia, que melhora após piscar o olho
- [ ] Outra: ____________________

14. TEM **SINTOMAS** OCULARES HABITUALMENTE NO **OLHO ESQUERDO**? *
(pode escolher várias opções)

Marcar tudo o que for aplicável.

- [ ] Não tenho sintomas
- [ ] Prurido (comichão)
- [ ] Picadelas
- [ ] Sensação de areias
- [ ] Sensação de Olho seco
- [ ] Sensação de visão turva
- [ ] Lacrimejo
- [ ] Olho vermelho
- [ ] Secreção
- [ ] Fotofobia (grande desconforto com a luz)
- [ ] Visão turva em certos momentos do dia, que melhora após piscar o olho
- [ ] Outra: ____________________

15. HISTÓRIA DE **DOENÇAS** OCULARES NO **OLHO DIREITO** *
(pode escolher várias opções)

Marcar tudo o que for aplicável.

☐ Nenhuma
☐ Olho seco
☐ Blefarite (inflamação das pálpebras)
☐ Glaucoma (ou história de hipertensão ocular)
☐ Retinopatia diabética
☐ Uveítes (inflamação intraocular)
☐ Outra: ______________________

16. HISTÓRIA DE **DOENÇAS** OCULARES NO **OLHO ESQUERDO** *
(pode escolher várias opções)

Marcar tudo o que for aplicável.

☐ Nenhuma
☐ Olho seco
☐ Blefarite (inflamação das pálpebras)
☐ Glaucoma (ou história de hipertensão ocular)
☐ Retinopatia diabética
☐ Uveítes (inflamação intraocular)
☐ Outra: ______________________

17. FAZ ALGUM **COLÍRIO** NO **OLHO DIREITO**? (gotas oculares) *

Marcar apenas uma oval.

◯ Sim
◯ Não

18. Se **SIM** na pergunta anterior, descreva **qual(s)**

____
____
____
____
____

19. FAZ ALGUM **COLÍRIO** NO **OLHO ESQUERDO**? (gotas oculares) *

Marcar apenas uma oval.

- Sim
- Não

20. Se **SIM** na pergunta anterior, descreva **qual(s)**

____
____
____
____
____

21. HISTÓRIA DE **CIRURGIA** OCULAR NO **OLHO DIREITO** *

Marcar tudo o que for aplicável.

- [ ] Nenhuma
- [ ] Cirurgia de Catarata
- [ ] Cirurgia de correção da miopia LASER
- [ ] Cirurgia de correção da miopia com Lentes Intraoculares
- [ ] Cirurgia de Glaucoma
- [ ] Vitrectomia (por descolamento de retina, buraco macular, membrana epirretiniana ou outra causa)
- [ ] Cirurgia de Explante (cirurgia extraocular após descolamento de retina)
- [ ] Cirurgia de correção de Estrabismo
- [ ] Cirurgia da Pálpebra, Órbita ou Vias Lacrimais
- [ ] Outra: ____________________

22. HISTÓRIA DE **CIRURGIA** OCULAR NO **OLHO ESQUERDO** *

Marcar tudo o que for aplicável.

- [ ] Nenhuma
- [ ] Cirurgia de Catarata
- [ ] Cirurgia de correção da miopia, hipermetropia ou astigmatismo LASER
- [ ] Cirurgia de correção da miopia, hipermetropia ou astigmatismo com Lentes Intraoculares
- [ ] Cirurgia de Glaucoma
- [ ] Vitrectomia (por descolamento de retina, buraco macular, membrana epirretiniana ou outra causa)
- [ ] Cirurgia de Explante (cirurgia extraocular após descolamento de retina)
- [ ] Cirurgia de correção de Estrabismo
- [ ] Cirurgia da superficie ocular e conjuntiva (Pterígeo ou Neoplasia)
- [ ] Cirurgia da Pálpebra, Órbita ou Vias Lacrimais
- [ ] Outra: ____________________

23. HISTÓRIA DE AMBLIOPIA **(olho preguiçoso)** *

Marcar apenas uma oval.

- Nenhum olho
- Olho direito
- Olho esquerdo
- Ambos os olhos

24. HISTÓRIA DE **TRAUMATISMO** OCULAR *

Marcar apenas uma oval.

- Nenhum olho
- Olho direito
- Olho esquerdo
- Ambos os olhos

25. HISTÓRIA DE **ERRO REFRATIVO** *

Marcar tudo o que for aplicável.

- [ ] Nenhum
- [ ] Miopia
- [ ] Hipermetropia
- [ ] Astigmatismo
- [ ] Presbiopia (vista cansada para o perto)

26. HISTÓRIA **PESSOAL** DE **DOENÇAS ECTÁSICAS** DA **CÓRNEA** *

Marcar tudo o que for aplicável.

- [ ] Não
- [ ] Sim, suspeita ou diagnóstico de Queratocone (ou outra ectasia) no olho direito
- [ ] Sim, suspeita ou diagnóstico de Queratocone (ou outra ectasia) no olho esquerdo
- [ ] Sim, suspeita ou diagnóstico de Queratocone (ou outra ectasia) em ambos os olhos

27. HISTÓRIA **FAMILIAR** DE **DOENÇAS ECTÁSICAS** DA **CÓRNEA** (Queratocone ou outra ectasia) *

Marcar apenas uma oval.

- Sim
- Não

28. HISTÓRIA **PESSOAL** DE **OUTRAS** DOENÇAS DA **CÓRNEA** (ex.: distrofias) *

Marcar apenas uma oval.

- Não
- Sim, olho direito
- Sim, olho esquerdo
- Sim, ambos os olhos

29. Se **SIM** na pergunta anterior, descreva **qual(s)**

30. HISTÓRIA **FAMILIAR** DE **OUTRAS** DOENÇAS DA **CÓRNEA** (ex.: distrofias) *

Marcar apenas uma oval.

- Sim
- Não

31. Se **SIM** na pergunta anterior, descreva **qual(s)**

32. USO DE **ÓCULOS** GRADUADOS *

Marcar apenas uma oval.

- Não
- Olho direito
- Olho esquerdo
- Ambos os olhos

33. USO DE **LENTES DE CONTACTO** *

Marcar apenas uma oval.

- Não
- Olho direito
- Olho esquerdo
- Ambos os olhos

DADOS SOBRE **ESTILO DE VIDA**

34. QUANTAS **HORAS** POR **SEMANA** PASSA AO **AR LIVRE**? *

Marcar apenas uma oval.

- Nunca
- 1 a 5 horas
- 5 a 10 horas
- 10 a 15 horas
- 15 a 20 horas
- 20 a 25 horas
- 25 a 30 horas
- Mais de 30 horas

35. DURANTE O **TEMPO** QUE PASSA AO AR LIVRE, UTILIZA **ÓCULOS DE SOL**? *

Marcar apenas uma oval.

- Nunca
- Menos de metade do tempo
- Mais de metade do tempo

36. QUANTOS **DIAS** DE **PRAIA** (PISCINA OU SIMILAR) FAZ **POR ANO**, EM MÉDIA? *

Marcar apenas uma oval.

- Nunca
- 1 a 10 dias
- 10 a 20 dias
- 20 a 30 dias
- 30 a 40 dias
- 40 a 50 dias
- Mais de 50 dias

37. QUANTAS **HORAS** POR **SEMANA** PRATICA **ATIVIDADE FÍSICA**? *

Marcar apenas uma oval.

- Nunca
- 1 a 2 horas
- 3 a 4 horas
- 5 a 6 horas
- 7 a 8 horas
- 9 a 10 horas
- Mais de 10 horas

38. **QUAL(S)** A(S) **ATIVIDADES FÍSICAS** QUE PRATICA? (pode escolher várias opções) *

Marcar tudo o que for aplicável.

- Caminhada
- Corrida
- Desportos de contacto (Boxe, KickBoxing, Jiu Jitsu, Taekondo, Karaté, Krav Maga)
- CrossFit
- Ginásio com Pesos / Halterofilismo
- Desportos coletivos (Futebol, Andebol, Basquetebol, Voleibol)
- Natação
- Ciclismo
- Outra: ______________________

39. CONTACTA **DIARIAMENTE** COM **AR CONDICIONADO** EM CASA OU NO LOCAL DE TRABALHO? *

Marcar apenas uma oval.

- Sim
- Não

40. A SUA **ATIVIDADE PROFISSIONAL** INCLUI A **UTILIZAÇÃO** DE **COMPUTADOR** OU OUTRO TIPO DE **ECRÃNS**? *

Marcar apenas uma oval.

- Sim
- Não

41. QUANTAS **HORAS** POR **DIA** UTILIZA **ECRÃS**? *

Marcar apenas uma oval.

- Nunca
- 1 a 2 horas
- 3 a 4 horas
- 5 a 6 horas
- 7 a 8 horas
- 8 a 10 horas
- Mais de 10 horas

42. QUANTAS **HORAS** POR **DIA** PASSA A **LER OU ESCREVER**? *

Marcar apenas uma oval.

- Nunca
- 1 a 2 horas
- 3 a 4 horas
- 5 a 6 horas
- 7 a 8 horas
- 8 a 10 horas
- Mais de 10 horas

43. NA MAIORIA DOS DIAS, **COSTUMA DORMIR** DE **OLHOS ABERTOS**? *

Marcar apenas uma oval.

- Sim
- Não
- Não sei

44. NA MAIORIA DOS DIAS, **COSTUMA DORMIR** COM? *

Marcar apenas uma oval.

- Barriga para baixo
- Barriga para cima

45. NA MAIORIA DOS DIAS, **COSTUMA DORMIR** COM? *

Marcar apenas uma oval.

- Com o lado direito da cara na almofada
- Com o lado esquerdo da cara na almofada

46. COSTUMA **ESFREGAR/COÇAR** O OLHO **DIREITO** *

Marcar apenas uma oval.

- Não
- 1 a 5 vezes por dia
- 6 a 10 vezes por dia
- 11 a 15 vezes por dia
- 16 a 20 vezes por dia
- Mais de 20 vezes por dia

47. COSTUMA **ESFREGAR/COÇAR** O OLHO **ESQUERDO** *

Marcar apenas uma oval.

- Não
- 1 a 5 vezes por dia
- 6 a 10 vezes por dia
- 11 a 15 vezes por dia
- 16 a 20 vezes por dia
- Mais de 20 vezes por dia

Este conteúdo não foi criado nem aprovado pela Google.

Google Formulários
